# A Low-Level Carbon Dioxide Laser Promotes Fibroblast Proliferation and Migration through Activation of Akt, ERK, and JNK

**DOI:** 10.1371/journal.pone.0168937

**Published:** 2017-01-03

**Authors:** Yoshiaki Shingyochi, Shigeyuki Kanazawa, Satoshi Tajima, Rica Tanaka, Hiroshi Mizuno, Morikuni Tobita

**Affiliations:** Department of Plastic and Reconstructive Surgery, Juntendo University School of Medicine, Hongo, Bunkyo-ku, Tokyo, Japan; Massachusetts General Hospital, UNITED STATES

## Abstract

**Background:**

Low-level laser therapy (LLLT) with various types of lasers promotes fibroblast proliferation and migration during the process of wound healing. Although LLLT with a carbon dioxide (CO_2_) laser was also reported to promote wound healing, the underlying mechanisms at the cellular level have not been previously described. Herein, we investigated the effect of LLLT with a CO_2_ laser on fibroblast proliferation and migration.

**Materials and Methods:**

Cultured human dermal fibroblasts were prepared. MTS and cell migration assays were performed with fibroblasts after LLLT with a CO_2_ laser at various doses (0.1, 0.5, 1.0, 2.0, or 5.0 J/cm^2^) to observe the effects of LLLT with a CO_2_ laser on the proliferation and migration of fibroblasts. The non-irradiated group served as the control. Moreover, western blot analysis was performed using fibroblasts after LLLT with a CO_2_ laser to analyze changes in the activities of Akt, extracellular signal-regulated kinase (ERK), and Jun N-terminal kinase (JNK), which are signaling molecules associated with cell proliferation and migration. Finally, the MTS assay, a cell migration assay, and western blot analysis were performed using fibroblasts treated with inhibitors of Akt, ERK, or JNK before LLLT with a CO_2_ laser.

**Results:**

In MTS and cell migration assays, fibroblast proliferation and migration were promoted after LLLT with a CO_2_ laser at 1.0 J/cm^2^. Western blot analysis revealed that Akt, ERK, and JNK activities were promoted in fibroblasts after LLLT with a CO_2_ laser at 1.0 J/cm^2^. Moreover, inhibition of Akt, ERK, or JNK significantly blocked fibroblast proliferation and migration.

**Conclusions:**

These findings suggested that LLLT with a CO_2_ laser would accelerate wound healing by promoting the proliferation and migration of fibroblasts. Activation of Akt, ERK, and JNK was essential for CO_2_ laser-induced proliferation and migration of fibroblasts.

## Introduction

Wound healing is a complex biological process that involves a cascade of events, including blood coagulation, inflammation, new tissue formation, and tissue remodeling. This process requires the collaborative efforts of several cell types such as keratinocytes, fibroblasts, endothelial cells, and immune cells [1, 2]. Cell migration, proliferation, differentiation, and extracellular matrix deposition are activated during wound healing. In particular, the proliferation and migration of fibroblasts play crucial roles in the formation of granulation tissue and lead to wound closure. During fibroblast migration and proliferation, several intracellular and intercellular pathways are activated and coordinated [3–5].

Recent studies showed that low-level laser therapy (LLLT) with various types of lasers promotes wound healing through tissue repair and reduces inflammation. The most common methods of irradiation in LLLT include lasers such as helium neon (He-Ne; 632.8 nm), ruby (694 nm), argon (488 and 514 nm), krypton (521, 530, 568, and 647 nm), gallium-aluminum-arsenide (805 and 650 nm), and gallium arsenide (904 nm) [6–8]. In the term of LLLT, several terms were introduced such as Photobiomodulation (PBM) therapy, Biostimulation, Cold/Cool Laser, Soft Laser and Low Power Laser Therapy.

Meanwhile, the carbon dioxide (CO_2_) laser is a long pulsed infrared laser with a wavelength of 10,600 nm, which is absorbed strongly by water [9]. A high-power CO_2_ laser is widely used in a variety of surgical procedures, including oral surgery and dermatologic surgery, as an alternative to a traditional scalpel [10, 11]. Recent studies focused on oral fibroblasts in the dental field also reported that LLLT with a CO_2_ laser promotes wound healing [12–16]. In basic research of LLLT with a CO_2_ laser for wound healing, Kenneth et al. showed that a super-pulsed CO_2_ laser decreases transforming growth factor-β1 secretion and increases basic fibroblast growth factor secretion of both normal and keloid dermal fibroblasts in vitro, and, as a result, promotes cell replication and may provide the ability to balance collagen organization against fibrosis [17]. Furthermore, regarding the role of fibroblasts in the process of wound healing, LLLT with other lasers promotes both proliferation and migration [18–23]. Moreover, LLLT activates fibroblast signaling pathways such as the extracellular signal-regulated kinase (ERK)/FOXM1 pathway [24].

However, the effects of LLLT with a CO_2_ laser on dermal fibroblast proliferation, migration, and signaling pathways at the cellular level are still not clearly understood.

In the present study, we investigated the effects of LLLT with a CO_2_ laser on dermal fibroblast proliferation and migration, and examined the involvement of the Akt, ERK, and Jun N-terminal kinase (JNK) pathways in dermal fibroblast proliferation and migration.

## Materials and Methods

### Cell culture

Human dermal fibroblasts (HDFs) (American Type Culture Collection, VA, USA) were prepared. HDFs (3.0 × 10^5^ cells) were cultured on a 100 mm dish in Dulbecco’s modified Eagle medium (DMEM) (Gibco BRL, MI, USA) containing 10% fetal bovine serum (FBS) (Gibco BRL), 100 U/mL penicillin, and 100 mg/mL streptomycin (Wako, Tokyo, Japan) in a humidified incubator at 37°C with a 5% CO_2_ atmosphere.

### CO_2_ laser irradiation

**Fig 1:**
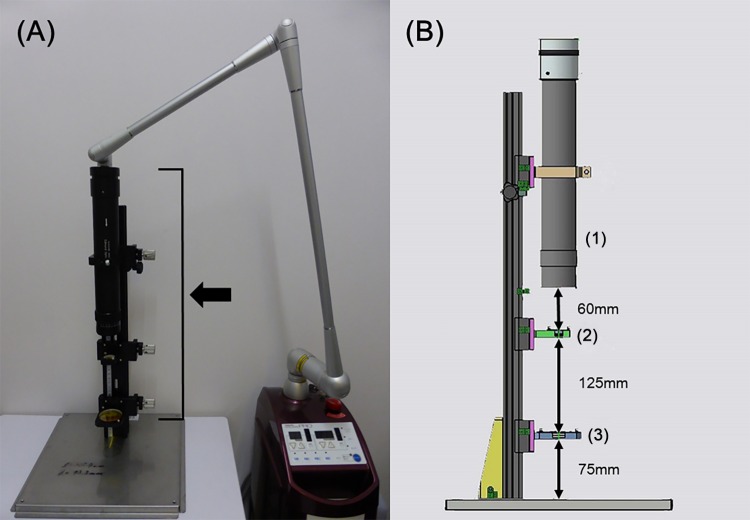
The CO_2_ laser machine equipped with a homogenizer. (A) The CO_2_ laser system was equipped with a homogenizer (arrow) attached to the end of the articulated arm in order to equalize the profile of the laser beam. Power densities were generated in a round homogeneous spot with a diameter of 35 mm. (B) The homogenizer was composed of (1) the beam shaper, (2) the aspherical lens (a diameter of 25.4 mm) and (3) the plano-convex lens (a diameter of 50.8 mm). The distance from the beam shaper to the aspherical lens was 60 mm, the aspherical lens to the plano-convex lens was 125 mm, and the plano-convex lens to the samples was 70 mm.

A CO_2_ laser system (Opelaser Pro, Yoshida Dental Mfg., Tokyo, Japan) operating at a wavelength of 10.6 μm was used. The CO_2_ laser system was equipped with a homogenizer attached to the end of the articulated arm in order to equalize the profile of the laser beam. Power densities were generated in a round homogeneous spot with a diameter of 35 mm (Fig 1). The CO_2_ laser system was used with continuous wave mode and following irradiation power (irradiance, irradiation time); 0.1 J/cm^2^ (52.08 mW/cm^2^, 2 sec), 0.5 J/cm^2^ (52.08 mW/cm^2^, 10 sec), 1.0 J/cm^2^ (52.08 mW/cm^2^, 20 sec), 2.0 J/cm^2^ (52.08 mW/cm^2^, 40 sec), and 5.0 J/cm^2^ (520.83 mW/cm^2^, 10 sec).

### Cell proliferation after LLLT

Cell proliferation was examined by 3-(4,5-dimethylthiazol-2-yl)-5-(3-carboxymethoxyphenyl)-2-(4-sulfophenyl)-2H-tetrazolium (MTS) assay (CellTiter 96^®^ Aqueous One Solution Cell Proliferation Assay, Promega, WI, USA). HDFs at passage 3–5 were plated at a density of 2000 cells per well in 96-well plates, incubated in DMEM containing 10% FBS for 24 h, and then incubated in DMEM containing 1.0% FBS for 24 h. After 48 h cells were plated, HDFs were washed twice with phosphate-buffered saline (PBS), which was aspirated before laser irradiation. HDFs in each well were evenly irradiated at an irradiation power of 0.1, 0.5, 1.0, 2.0, or 5.0 J/cm^2^ in continuous wave mode at 25°C (room temperature). The non-irradiated group served as the control. HDFs were incubated in DMEM containing 1.0% FBS for 48 h. Thereafter, 20 μL of CellTiter 96^®^ One Solution Reagent was added to each well of the 96-well assay plate containing HDFs in 100 μL of culture medium. HDFs were incubated for an additional 2 h at 37°C in a 5% CO_2_ atmosphere. The production of formazan by viable HDFs was measured as absorbance at 490 nm using a 96-well plate reader. To observe the effects of Akt, ERK, or JNK inhibition, HDFs were treated with an inhibitor of each signaling molecule, namely, 10 mM LY294002 (Jena Bioscience, Berlin, Germany), 10 mM U-0126 (Calbiochem, CA, USA), or 10 mM SP600125 (Calbiochem), respectively, for 60 min before irradiation. After treatment with each inhibitor, the MTS assay was conducted. Nine replicate samples were prepared in each assay. And the assay was repeated three times.

### Cell migration after LLLT

HDFs were plated at a density of 8000 cells per well in 96-well plates, incubated in DMEM containing 10% FBS for 24 h, and then incubated in DMEM containing 1.0% FBS for 24 h. Confluent HDFs were wounded using the WoundMaker device (Essen BioScience, MI, USA). Then, HDFs were washed twice with PBS, which was aspirated before laser irradiation. Thereafter, HDFs were irradiated under the same conditions as described for the MTS assay. Images of the wounded cell monolayers were monitored and quantified with the IncuCyte live-cell imager (Essen BioScience) at 0, 6, 12, 18, and 24 h after wounding. The migration rate was expressed as migration distance/time (μm/h). In addition, HDFs were treated with the inhibitors of each signaling molecule (LY294002, U-0126, or SP600125 [10 mM]) for 60 min before wounding. After each inhibitor treatment, the treated HDFs were wounded and monitored for 24 h. Nine replicate samples were prepared in each assay. And the assay was repeated three times.

### Western blot analysis

HDFs were plated at a density of 2.0 × 10^5^ cells per 100mm dish, incubated in DMEM containing 10% FBS for 24 h, and then incubated in DMEM containing 1.0% FBS for 24 h. Confluent HDFs were washed twice with PBS, which was aspirated before laser irradiation, and then irradiated with the laser at 1.0 J/cm^2^. At specified time points after irradiation (5, 15, or 30 min), HDFs were lysed in RIPA buffer, which contained 100 mM Tris, 150 mM NaCl, 0.1% sodium dodecyl sulfate, 0.5% deoxycholic acid sodium salt monohydrate (Nacalai Tesque, Kyoto, Japan), and 1.0% Nonidet P-40 (Wako, Osaka, Japan), incubated for 20 min at 4°C, and centrifuged at 15,000 × g for 15 min at 4°C. The proteins were separated by sodium dodecyl sulfate polyacrylamide gel electrophoresis and transferred onto nitrocellulose membrane using an iBlot 2 Dry Blotting System (Invitrogen, MA, USA). The membranes were incubated in Tris-buffered saline containing 5% skimmed milk and 0.05% Tween-20 for 60 min and blotted with primary antibodies at 4°C overnight. Anti-p-Akt (Ser473, 1:1000; Cell Signaling Technology, MA, USA), anti-Akt (1:1000; Cell Signaling Technology), anti-p-ERK (Thr202/Tyr204, 1:1000; Cell Signaling Technology), anti-ERK (1:1000; Cell Signaling Technology), anti-p-stress-activated protein kinase (SAPK)/JNK (Thr183/Tyr185, 1:1000; Cell Signaling Technology), anti-SAPK/JNK (1:1000; Cell Signaling Technology), and anti-GAPDH (1:2000; Abcam, Cambridge, UK) antibodies were used as primary antibodies. The membranes were incubated for 1 h with an anti-mouse or anti-rabbit horseradish peroxidase-linked secondary antibody (1:10,000; Cell Signaling Technology). Reaction products were visualized by detection of chemiluminescence using ImmunoStar LD (Wako, Osaka, Japan). Relative band densities were quantified using Multi Gauge software (Fujifilm, Tokyo, Japan). For inhibitor studies, HDFs were treated with the inhibitors of each signaling molecule (LY294002, U-0126, or SP600125 [10 mM]) for 60 min before irradiation. After each inhibitor treatment, western blot analyses were conducted using the treated HDFs. Three replicate samples were prepared in each assay. And the assay was repeated three times.

### Statistical analysis

Statistical analyses were performed using GraphPad Prism6 software (Graphpad software). All data are presented as the mean ± standard deviation. One-way ANOVA with unpaired samples was used to determine whether there are any statistically significant differences between the groups. A Tukey-Kramer test was then performed to compare groups. Significance was considered at p < 0.05.

## Results

### LLLT with a CO_2_ laser stimulated HDF proliferation

**Fig 2:**
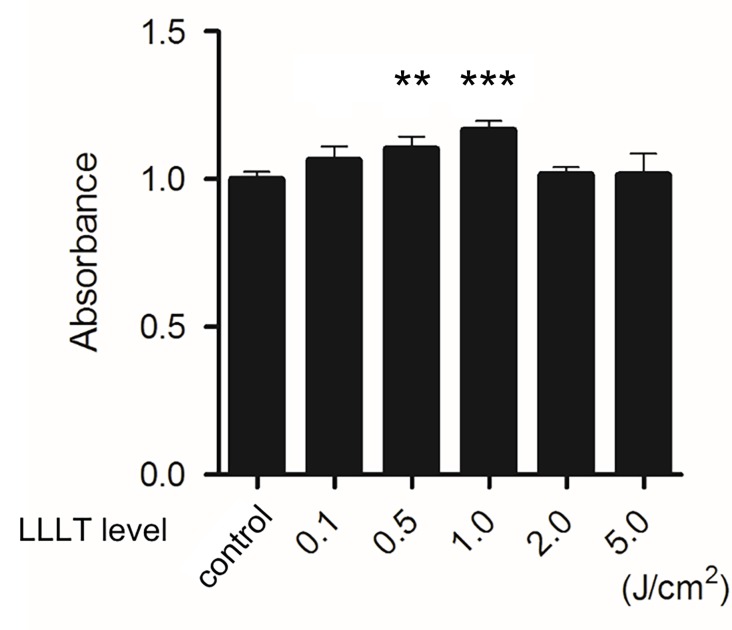
Effects of LLLT with a CO_2_ laser on HDF proliferation. HDF proliferation was determined by the MTS assay after irradiation with LLLT (0.1, 0.5, 1.0, 2.0, or 5.0 J/cm^2^). The non-irradiated group served as the control. Results are expressed as the mean ± SD of three independent experiments. **p<0.01, ***p<0.001 compared with the non-irradiated group (Tukey-Kramer test).

HDFs were irradiated under various conditions (0.1, 0.5, 1.0, 2.0, or 5.0 J/cm^2^) and incubated for 48 h. The non-irradiated group served as the control. Cell viability was then assessed with the MTS assay. LLLT with a CO_2_ laser statistically significantly promoted the proliferation of HDFs at doses of 0.5, and 1.0 J/cm^2^. LLLT with a CO_2_ laser at a dose of 1.0 J/cm^2^ most effectively promoted cell proliferation in this study (Fig 2).

### LLLT with a CO_2_ laser promoted HDF migration

**Fig 3:**
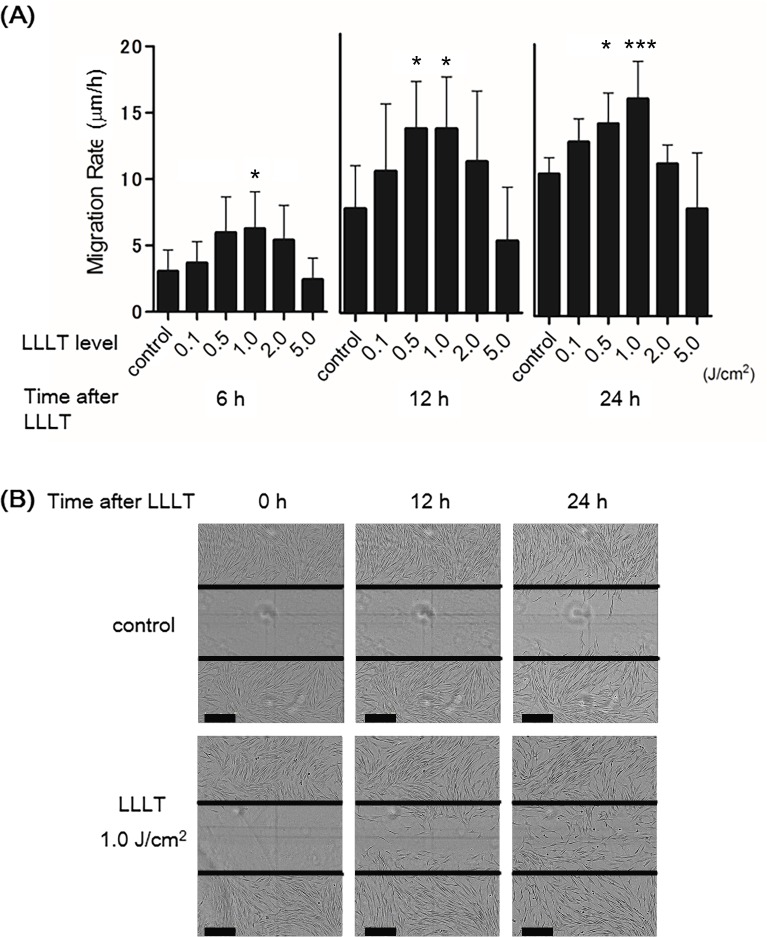
Effects of LLLT with a CO_2_ laser on HDF migration. A cell migration assay of HDFs treated with LLLT under several irradiation powers (0.1, 0.5, 1.0, 2.0, or 5.0 J/cm^2^). The non-irradiated group served as the control. (A) Migration rates (0.1, 0.5, 1.0, 2.0, or 5.0 J/cm^2^) are expressed as migration distance/time (μm/h). The non-irradiated group served as the control. Results are expressed as the mean ± SD of three independent experiments. *p<0.05, ***p<0.001 compared with the non-irradiated group (Tukey-Kramer test). (B) Images show the wounded cell monolayers at 0, 12, and 24 h after wounding with non-irradiated or irradiated (1.0 J/cm^2^) HDFs. The line indicates the wound edge at the start of the experiment (0 h). Bar = 300 nm. The migration of irradiated HDFs was promoted compared with the control.

To examine the effect of LLLT with a CO_2_ laser on dermal fibroblast migration, we monitored wounded HDFs for 24 h upon irradiation with various doses (0.1, 0.5, 1.0, 2.0, or 5.0 J/cm^2^). The non-irradiated group served as the control. Irradiated (0.5 and 1.0 J/cm^2^) HDFs showed a statistically significant increase in the migration rate at 24 h (14.13363095 and 15.97625 μm/h, respectively) (Fig 3A). Representative images demonstrated that the migration of irradiated (1.0 J/cm^2^) HDFs to the site of wounded cell monolayers was promoted at 12 and 24 h compared with the non-irradiated group (Fig 3B).

### Activation of Akt, ERK, and JNK was involved in CO_2_ laser-induced cell proliferation and migration

To examine the molecular mechanisms responsible for the effects of LLLT on fibroblasts, we investigated the involvement of Akt, ERK, and JNK in LLLT-induced fibroblast proliferation and migration. First, we measured the activation of these signaling molecules in response to LLLT stimulation using western blot analysis. Before western blotting, HDFs were irradiated with a CO_2_ laser at 1.0 J/cm^2^ and incubated for different durations (0–30 min). Second, we performed MTS and cell migration assays with LY294002, U-0126, or SP600125 (an Akt, ERK, and JNK inhibitor, respectively) to examine how inhibition of these proteins affects fibroblast proliferation and migration.

**Fig 4:**
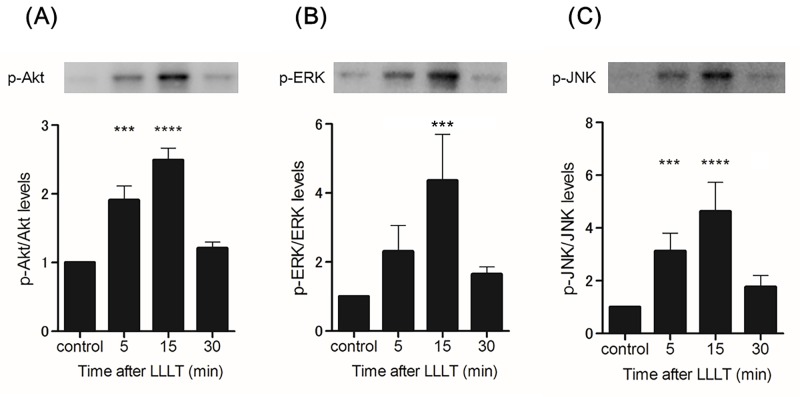
Effects of LLLT with a CO_2_ laser on the activities of Akt, ERK, and JNK. (A) Akt activity was analyzed by immunoblotting at 5, 15, and 30 min after LLLT at 1.0 J/cm^2^. Densitometric measurements of p-Akt were normalized to the amount of total Akt. (B) ERK activity was analyzed by immunoblotting at 5, 15, and 30 min after LLLT at 1.0 J/cm^2^. Densitometric measurements of p-ERK were normalized to the amount of total ERK. (C) JNK activity was analyzed by immunoblotting at 5, 15, and 30 min after LLLT at 1.0 J/cm^2^. Densitometric measurements of p-JNK were normalized to the amount of total JNK. Results are expressed as the mean ± SD of three independent experiments. ***p<0.001, ****p<0.0001 compared with the non-irradiated group as the control (Tukey-Kramer test).

**Fig 5:**
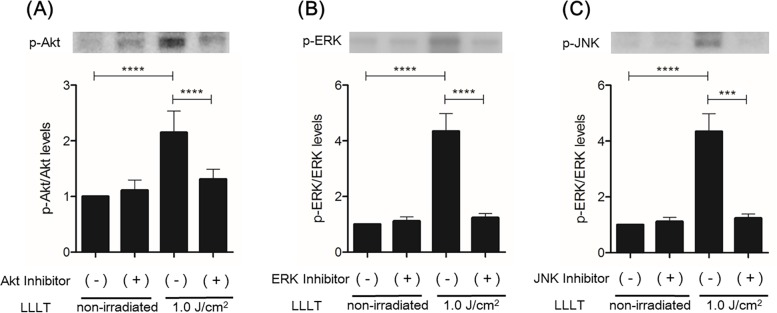
Effect of inhibition of Akt, ERK, or JNK on LLLT-induced activation of signaling molecules in HDFs. (A) Akt activity in HDFs treated with or without the inhibitor of Akt was analyzed by immunoblotting at 15 min after LLLT (1.0 J/cm^2^). Densitometric measurement of p-Akt was normalized to the amount of total Akt. (B) ERK activity in HDFs treated with or without the inhibitor of ERK was analyzed by immunoblotting at 15 min after LLLT (1.0 J/cm^2^). Densitometric measurement of p-ERK was normalized to the amount of total ERK. (C) JNK activity in HDFs treated with or without the inhibitor of JNK was analyzed by immunoblotting at 15 min after LLLT (1.0 J/cm^2^). Densitometric measurement of p-JNK was normalized to the amount of total JNK. The results are presented as fold change compared with the non-inhibited group. Results are expressed as the mean ± SD of three independent experiments. ***p<0.001, ****p<0.0001 (Tukey-Kramer test).

**Fig 6:**
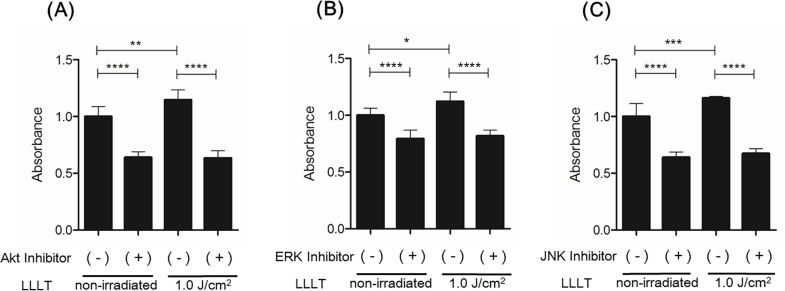
Effect of inhibition of Akt, ERK, or JNK on LLLT-induced HDF proliferation. (A) HDF proliferation was measured by the MTS assay after pretreatment with the inhibitor of Akt and subsequent LLLT irradiation (1.0 J/cm^2^). (B) HDF proliferation assay after pretreatment with the inhibitor of ERK and subsequent LLLT irradiation (1.0 J/cm^2^). (C) HDF proliferation assay after pretreatment with the inhibitor of JNK and subsequent LLLT irradiation (1.0 J/cm^2^). Results are expressed as the mean ± SD of three independent experiments. *p<0.05, **p<0.01, ***p<0.001, ****p<0.0001 (Tukey-Kramer test).

**Fig 7:**
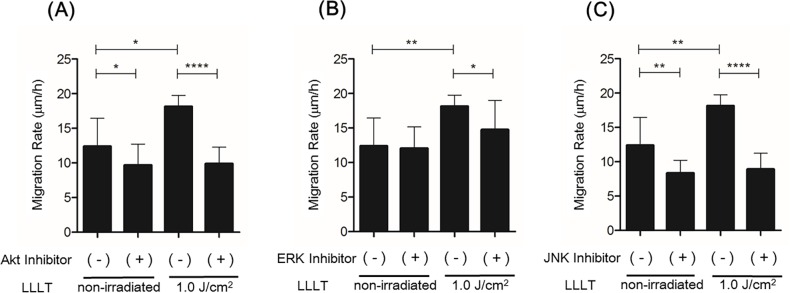
Effect of inhibition of Akt, ERK, or JNK on LLLT-induced HDF migration. (A) Cell migration assay of HDFs with or without LLLT irradiation (1.0 J/cm^2^) in the presence or absence of the inhibitor of Akt. The migration rate is expressed as migration distance/time (μm/h). (B) Cell migration assay of HDFs with or without LLLT irradiation (1.0 J/cm^2^) in the presence or absence of the inhibitor of ERK. (C) Cell migration assay of HDFs with or without LLLT irradiation (1.0 J/cm^2^) in the presence or absence of the inhibitor of JNK. Results are expressed as the mean ± SD of three independent experiments. *p<0.05, **p<0.01, ****p<0.0001 (Tukey-Kramer test).

LLLT stimulation significantly increased levels of p-Akt and p-JNK at 5–15 min and of p-ERK at 15 min (Fig 4). LY294002 significantly inhibited CO_2_ laser-induced Akt phosphorylation, U-0126 significantly inhibited CO_2_ laser-induced ERK phosphorylation, and SP600125 significantly inhibited CO_2_ laser-induced JNK phosphorylation (Fig 5). In the MTS assay, Akt-inhibited and irradiated (1.0 J/cm^2^) HDFs showed a statistically significant reduction in proliferation after 48 h of incubation compared with irradiated HDFs without Akt inhibition. ERK-inhibited HDFs and JNK-inhibited HDFs showed similar results (Fig 6). In the cell migration assay, Akt-inhibited and irradiated (1.0 J/cm^2^) HDFs showed a statistically significant reduction in migration at 24 h (10.48365 μm/h) compared with irradiated HDFs without Akt inhibition (18.12677222 μm/h). ERK-inhibited and irradiated (1.0 J/cm^2^) HDFs showed a statistically significant reduction in migration at 24 h (14.74557857 μm/h) compared with irradiated HDFs without ERK inhibition (18.12677222 μm/h). JNK-inhibited and irradiated (1.0 J/cm^2^) HDFs showed a statistically significant reduction in migration at 24 h (8.905873016 μm/h) compared with irradiated HDFs without JNK inhibition (18.12677222 μm/h) (Fig 7.) Thus, inhibition of Akt, ERK, and JNK significantly blocked CO_2_ laser-induced cell proliferation and migration.

## Discussion

High- or low-power laser therapies are used for therapeutic purposes. Low-power (non-surgical) lasers, which are defined as LLLT, are widely used to promote granulation and improve wound repair [25, 26]. In addition, they also have anti-inflammatory and analgesic effects [27, 28]. Although LLLT does not have an ablative or thermal mechanism like other medical laser procedures, a photochemical effect causes chemical changes in several tissues [6]. Mester et al. first reported LLLT as a therapeutic modality, showing that low-energy (1 J/cm^2^) irradiation with a ruby laser promotes wound healing [29–31]. Many reports suggest that LLLT such as gallium arsenide, He-Ne, argon, and ruby lasers, as well as a red light-emitting diode, stimulate wound healing [32–36]. Consistent with this, LLLT with a CO_2_ laser induces proliferation of fibrochondrocytes and gingival fibroblasts [15, 37]. However, the mechanisms of action underlying the effects of LLLT with a CO_2_ laser on skin wound healing are not clearly understood [17, 38–40].

Wound healing is a dynamic and complex biological process. Proliferation and migration of dermal fibroblasts have important roles in skin wound repair. Fibroblasts proliferate and migrate to the wound area, compose the new extracellular matrix, and conduce wound healing [4]. In this study, we focused on the activation of dermal fibroblasts and performed experiments to evaluate the wound healing effect of LLLT with a CO_2_ laser in vitro.

First, we demonstrated that HDFs irradiated with a CO_2_ laser at various power levels exhibited increased proliferation and migration. Treatment with a CO_2_ laser at 1.0 J/cm^2^ promoted fibroblast proliferation the most. Similar results were obtained for fibroblast migration. These data indicate that CO_2_ laser irradiation at 1.0 J/cm^2^ is most effective for fibroblast activation in this experiment. From these results, we adopted an irradiation power of 1.0 J/cm^2^ for subsequent western blotting to analyze the activation of various signaling molecules.

Akt is an important signaling factor for cell survival, proliferation, and migration [41–47]. In the present study, we demonstrated that LLLT with a CO_2_ laser-induced activation of Akt signaling and promoted fibroblast proliferation and migration. These findings suggested that Akt is an important factor for CO_2_ laser-induced fibroblast activation because the effects of the CO_2_ laser were inhibited after Akt signaling inhibition.

The MAPK family consists mainly of ERK, JNK, and p38 MAPK. ERK is implicated in the regulation of various cellular processes including cell proliferation, migration, growth, differentiation, and tumor progression [47–49]. The JNK pathway is activated by the exposure of cells to several stresses such as heat shock, cytokines, osmotic shock, protein synthesis inhibitors, oxidative stress, ultraviolet radiation, and DNA-damaging agents [50]. JNK is also involved in many cellular processes [49].

This study demonstrated that LLLT with a CO_2_ laser activated ERK and JNK. Furthermore, their inhibition significantly blocked CO_2_ laser-induced cell proliferation and migration. These results suggested that CO_2_ laser-induced fibroblast proliferation and migration also require ERK or JNK activation.

These findings are in line with previous research showing that LLLT with several lasers activates the signaling molecules. Although expression of signaling molecules such as MAPKs differs among different cell types, various studies have shown a correlation between cell proliferation and MAPK stimulation as a reaction to extracellular stimuli [50]. Miyata et al. reported that MAPK/ERK plays a role in the increased proliferation of human dental pulp cells following low-level diode laser irradiation [51]. Furthermore, LLLT with a He-Ne laser stimulates Akt activation, which is mediated by PI3K, and activation of the PI3K/Akt signaling pathway is crucial for promoting cell proliferation and migration induced by LLLT [52, 53].

A CO_2_ laser is one of the most commonly used medical lasers, especially in oral surgery and skin surgery, because it is inexpensive compared with other medical lasers and is easy to use. To our knowledge, the present study is the first to investigate the activation of signaling mechanisms in dermal fibroblasts upon LLLT with a CO_2_ laser. However, further research is required to elucidate the mechanisms upstream or downstream of Akt, ERK, and JNK activation by LLLT with a CO_2_ laser.

It is not clear how LLLT with a CO_2_ laser activates the signaling molecules. Some reports suggest that intracellular photobiostimulation of LLLT occurs via the electron transport chain enzymes in mitochondria, which increases cellular metabolism and function [54, 55]. The photonic energy is converted to chemical energy in the form of ATP, which enhances cellular functions [56]. However, additional work is needed to elucidate how these mechanisms lead to activation of the signaling molecules.

In conclusion, the present study demonstrated that LLLT with a CO_2_ laser mediates HDF proliferation and migration by the activation of Akt, ERK, and JNK. Our findings provide novel mechanistic insights into the positive effects of LLLT with a CO_2_ laser on fibroblast proliferation and migration.
